# Establishment of a mouse model of respiratory mycoplasma challenged intranasally combined intraperitoneally eliciting chronic inflammation and lung consolidation

**DOI:** 10.1016/j.gendis.2024.101243

**Published:** 2024-02-09

**Authors:** Xing Xie, Yuan Gan, Maoda Pang, Lei Zhang, Fei Hao, Yanna Wei, Yi Chen, Rong Chen, Zhenzhen Zhang, Qingyun Xie, Daesub Song, Guoqing Shao, Maojun Liu, Qiyan Xiong, Zhixin Feng

**Affiliations:** a Key Laboratory for Veterinary Bio-Product Engineering, Ministry of Agriculture and Rural Affairs, Institute of Veterinary Medicine, Jiangsu Academy of Agricultural Sciences, Nanjing, Jiangsu 210014, China; b Jiangsu Key Laboratory for Food Quality and Safety-State Key Laboratory Cultivation Base, Ministry of Science and Technology, Nanjing, Jiangsu 210014, China; c GuoTai (Taizhou) Center of Technology Innovation for Veterinary Biologicals, Taizhou, Jiangsu 225300, China; d Key Laboratory of Control Technology and Standard for Agro-product Safety and Quality, Key Laboratory of Food Quality and Safety of Jiangsu Province-State Key Laboratory Breeding Base, Institute of Food Safety and Nutrition, Jiangsu Academy of Agricultural Sciences, Nanjing, Jiangsu 210014, China; e College of Veterinary Medicine, Xinjiang Agricultural University, Urumqi, Xinjiang 830052, China; f College of Veterinary Medicine, Seoul National University, Seoul 08826, South Korea

*Mycoplasma hyopneumoniae* (Mhp) and *Mycoplasma ovipneumoniae* (Mo), similar to *Mycoplasma pneumoniae* (Mp), initiate a chronic respiratory infection with concomitant pulmonary disease, causing substantial harm worldwide.^1^ A mouse model with genetic consistency helped to elucidate the unknown pathogenic mechanism of respiratory mycoplasmas in large animals and humans. While the intraperitoneal route would facilitate dissemination of infectious agents to the adventitia, combined challenge or immunization would promote replication of microorganisms or vaccine immune effects; for example, intraperitoneal combined intranasal route increased gamma interferon-specific CD8 T cells by more than 60 times after influenza infection.^2^

Here, a BALB/c mouse model was constructed to assess the pathogenic potential of respiratory mycoplasmas and to determine whether the intranasal combined or without intraperitoneal challenge is a sufficient general method. Each mouse was inoculated twice intranasally combined or without intraperitoneally with virulent mycoplasma strains Mp 129, Mo IK3-3, Mhp JS (highly virulent), Mhp J, and Mhp 168 L (low virulence) at a final concentration of 10^9^ CCU_50_/mL or phosphate buffer saline (PBS) at 0 day and 2 days post inoculation (dpi). A flow chart of the infection and methods is shown in Figure 1A and File S1. Our mouse infection model was not lethal, as Mhp, Mp, and Mo infections are chronic and not lethal. Mice in groups Mhp JS, Mhp J, Mp, and Mo, especially by the intranasal and intraperitoneal route, showed progressive clinical symptoms, including ruffled fur, decreased activity, and decreased appetite. The body weights of mice in the PBS and 168 L groups gradually increased by over 20%, while those in the JS and J groups increased by only 5% and 10%, respectively (Fig. S1). Cough was not observed, but the pleural skin trembling phenomenon was observed in the Mp- and Mhp JS-infected groups challenged by the intranasal combined intraperitoneal route, and mice infected with virulent respiratory mycoplasmas intranasally plus intraperitoneally can produce representative dark reddish consolidation, and obvious lesions are highlighted by black arrows (Fig. 1B). Gross lung lesions appeared at 14 dpi, with the most significant degree at 21 dpi. No significant difference appeared between 21 and 28 dpi. Most importantly, at 35 dpi, gross lung lesions in some mice were captured. A single intranasal challenge could not induce gross lung lesions after Mhp infection; only one mouse inoculated with Mhp JS (1/6) and Mp (1/6) intranasally, while mice inoculated with Mhp JS (6/6) and Mp (6/6), Mhp J (2/6) and Mo (4/6) showed consolidation by the intranasal combined intraperitoneal route (Table S1).

**Figure 1:**
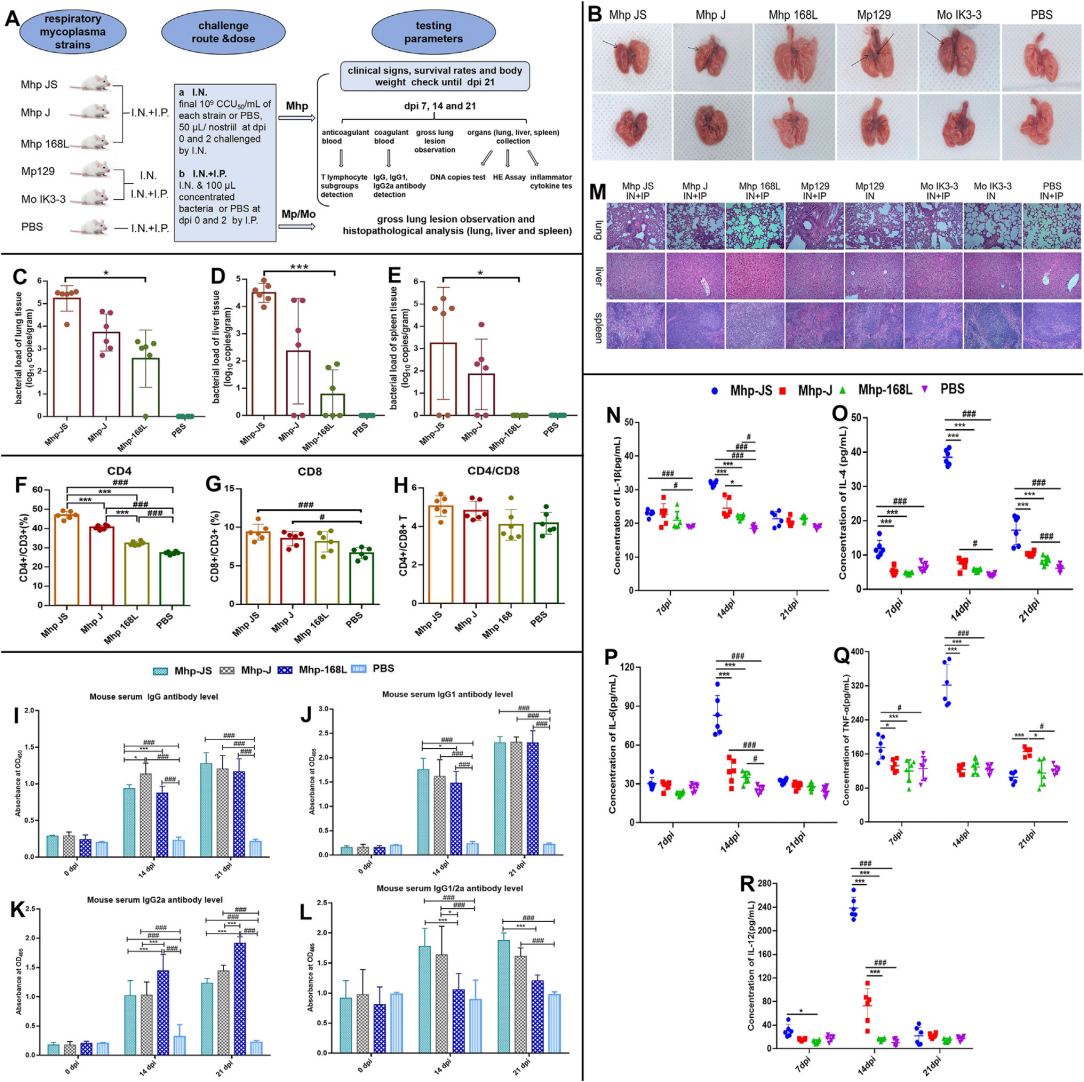
The establishment of a mouse infection pathogenesis and virulence evaluation model of *Mycoplasma hyopneumoniae* (Mhp) strains that can cause lung consolidation in mice by intranasal combined or without intraperitoneal injection. **(A)** Flow chart of the mouse Mhp infection experiment scheme. Every 17 five-week-old BALB/c mice were intranasally (I.N.) and intraperitoneally (I.P.) challenged twice with 10^9^ CCU_50_/mL JS, J, or 168 L strain or phosphate buffer saline (PBS) in 0.2 mL at 0 day and 2 days post inoculation (dpi). For the Mp or Mo challenged group, 12 mice per strain were randomly divided into two parts: half mice were I.N. inoculated with strains using the abovementioned dose, and the remaining mice were I.N. combined I.P. injected as described above. Five mice per group, distinguished by ear tags, were used for the evaluation of clinical signs, survival rate, and body weight. Anticoagulant blood from another six mice per group at 14 and 21 dpi was collected for T lymphocyte subgroup detection; coagulant blood was collected for IgG and subtype antibody detection; lungs were collected for gross lesion and histopathological scoring; and lung, liver, and spleen tissues were collected for qRT‒PCR, histopathological analysis, and inflammatory cytokine testing. **(B)** Gross lung lesion assessment at 21 dpi after necropsy. Whole lung samples connected to the trachea of each piglet were imaged after the animals were sacrificed. Gross lung lesions from BALB/c mice in the three Mhp strain-infected groups or mock-infected PBS control group inoculated by I.N. combined I.P method, and from Mp 129 and Mo IK3-3 strain-infected groups inoculated with I.N. combined/without I.P. challenge routes. Representative pictures of dorsal (Line 1) and ventral views (Line 2) were taken for assessment of gross lesions of the Mhp strain-infected groups at 21 dpi for JS, J, 168 L, Mp129, and Mo IK3-3. Macroscopic images of BALB/c mouse lungs in the PBS control group were also taken. **(C**–**E)** qRT‒PCR analysis of Mhp from various tissues of mice inoculated with various respiratory mycoplasmas and PBS. Homogenates of three tissues, including the lung (C), liver (D), and spleen (E), of mice were collected, and the DNA copies of P97 per gram of tissue were quantified. ∗*p* < 0.05 and ∗∗∗*p* < 0.01 indicate a significant difference in DNA copies per gram of lung tissues between the JS and 168 L groups. **(F–H)** Mean percentages of CD4^+^ and CD8^+^ T lymphocytes in mouse peripheral blood mononuclear cells (PBMCs) between three Mhp-infected groups and the PBS control group at 14 dpi. (F) Percentage of CD4^+^ and CD3^+^ T cells in PBMCs between the Mhp-infected and control groups. (G) Percentage of CD8^+^ and CD3^+^ T cells in PBMCs between the Mhp-infected and control groups. (H) Percentage of CD4^+^ and CD8^+^ T cells in PBMCs between the Mhp-infected and control groups. ∗∗∗*p* < 0.01 indicates a significant difference in the mean percentage of CD3^+^ CD4^+^ T lymphocytes between the JS and J groups, JS and 168 L groups, or between the J and 168 L groups. ^#^*p* < 0.05 and ^###^*p* < 0.01 indicate that the mean percentage of CD3^+^ CD4^+^ T lymphocytes in the JS-, J- and 168 L-infected groups was obviously higher than that in the PBS group, and also indicate a significant difference in the mean percentage of CD3^+^ CD8^+^ T lymphocytes between the JS and PBS groups or between the J and PBS groups. **(I**–**L)** Mhp-specific antibody titer secretion levels (IgG, IgG1, and IgG2a) in mouse serum in the infected and control groups. Serum anti-Mhp IgG titers included those of IgG (I), IgG1 (J), and IgG2a (K), and the ratio of IgG1/2a (L). Mice were assayed with ELISA coated with the whole-cell lysate proteins of Mhp strain 168. The cut-off value shown as OD_450_ or OD_405_ was calculated in terms of the mean OD of those at 0 dpi (mouse negative serum). S/N value = sample OD_450 or 405_/negative serum control OD_450 or 405_. S/N values higher than 2.1 were considered positive. ∗*p* < 0.05 and ∗∗∗*p* < 0.01 indicate significant differences in IgG titers at 14 dpi between the JS and J groups or between the JS and 168 L groups; IgG1 titers at 14 dpi between the JS and 168 L groups; IgG2a titers at 14 and 21 dpi between the JS and 168 L groups or between J and 168 L groups; and ratio of IgG1/2a at 14 dpi between the JS and 168 L groups or between the J and 168 L groups, and at 21 dpi between the JS and 168 L groups. ^#^*p* < 0.05 and ^###^*p* < 0.01 indicate that mouse serum-specific antibody IgG, IgG1, and IgG2a expression levels in the Mhp strain JS-, J-, and 168 L-infected groups were obviously higher than those in the PBS group at 14 and 21 dpi. **(M)** Histopathological changes in the lung, liver, and spleen of BALB/c mice infected with three Mhp strains through the I.N. combined I.P. challenge route and mice infected with Mp129 and Mo IK3-3 challenged I.N. with/without I.P. at 21 dpi. Lines 1–3 depict representative histopathological lesions of the lung and liver of spleen tissues from mice in the Mhp JS, J, 168 L, Mp 129-I.N.+I.P., Mp 129-I.N., Mo IK3-3-I.N.+I.P., Mo IK3-3-I.N., and PBS*-*inoculated groups at 100 × magnification. The histopathological pneumonia lesions from lung samples were evaluated following the criteria of lymphocyte infiltration and congestion or hemorrhage; the severity of the lesion was scored as 0 (normal), 1 (mild), 2 (moderate), and 3 (severe). **(N–R)** Proinflammatory cytokine production in mouse lung homogenates induced by Mhp at 7, 14, and 21 dpi. Proinflammatory cytokine secretion, including IL-1β (N), IL-4 (O), IL-6 (P), TNF-α (Q), and IL-12 (R), from lung tissue in the infected groups and PBS control group was determined using ELISA. ∗*p* < 0.05 and ∗∗∗*p* < 0.01 indicate significant differences in IL-1β, IL-4, TNF-α, and IL-12 secretion levels from lung homogenates between the JS and J groups or between the JS and 168 L groups at 7 dpi, and significant differences in all five cytokine secretion levels between the JS and J groups or between the JS and 168 L groups at 14 dpi, and significant differences in IL-4 and TNF-α secretion levels between the JS and J groups or between the JS and 168 L, or between J and 168 groups at 21 dpi. ^#^*p* < 0.05 and ^###^*p* < 0.01 indicate significant differences in five cytokine secretion levels between the JS and PBS groups or between the J and PBS groups at 14 and 21 dpi. Data were expressed as mean ± standard deviation from six independent replicates.

To establish evaluation criteria to distinguish Mhp virulence and the bacterial load of strain-infected lungs (100%, 6/6; Fig. 1C), average DNA copies in lungs were much higher than those in the livers and spleens (Fig. 1D, E; Table S2). DNA copies/gram of three tissues in the JS group were significantly higher than those in the 168 L group, with the lung having the highest bacterial load and detection rate (Table S2). The mean percentages of CD3^+^ CD4^+^ (Fig. 1F) and CD3^+^ CD8^+^ T lymphocytes (Fig. 1G) in the JS and J groups were significantly higher than those in the 168 L group. There was no significant difference between the infected and control groups with regard to the ratio of CD4/CD8 (Fig. 1H), consistent with a report on Mhp-infected pigs, in which helper (CD4^+^) T cells were more prevalent than cytotoxic (CD8^+^) T cells.^3^

Moreover, anti-Mhp IgG (Fig. 1I), IgG1 (Fig. 1J), and IgG2a titers (Fig. 1K) at 21 dpi all increased compared with those at 14 dpi. JS depicted significantly higher IgG than those in the J group and higher IgG1 values than those in the 168 L group. JS- and J-infected mice demonstrated significantly higher IgG2a values than those in the 168 L group. IgG, IgG1, and IgG2a titers in JS and J groups and IgG1/2a in JS group were significantly higher than those in PBS control groups. The ratio of IgG1/2a at both time points showed a similar pattern, and the ratio mean values in JS, J, and 168 L groups at 14 dpi were 1.776, 1.636, and 1.056, respectively, indicating that IgG1 is mainly involved in partial activation of humoral immune responses and that specific Th2 cell responses predominate.

Lung tissues inoculated with Mhp JS, J, and 168 L intranasally combined intraperitoneally, Mp and Mo by the intranasal combined or without intraperitoneal route showed histopathological lesions of interstitial pneumonia, the extent of which was positively correlated with intranasal combined or without intraperitoneal challenge route and Mhp strain virulence. Lungs in the Mhp JS and Mp groups by the intranasal combined intraperitoneal route showed the most severe lesions, including widened alveolar space, congestion, or hemorrhage. Lung histopathological scores in the Mo and Mp groups after two intranasal combined intraperitoneal challenges were obviously higher than those with two intranasal challenges (Fig. S2). We previously constructed an Mhp vaccine with PEG-grafted hybrid nanovesicles. After a 10-fold concentrated virulent Mhp strain was once intranasally injected into BALB/c mice, interstitial pneumonia appeared^4^; however, the strain and titer were both different; thus, a single intranasal challenge can only induce lung histopathology, not lung gross lesions.

Pathological lesions in tissues other than the lungs of pigs infected with Mhp have not been described. Here, degenerated cells of the liver and lymphocytes of the spleen were markedly absent (Fig. 1M). Mice challenged with virulent respiratory mycoplasma strains by the intranasal combined intraperitoneal route showed degeneration of blisters differing in severity in the liver and splenic infarction in the spleen. Lesions in the Mhp group were the most severe, and those in the Mp and Mo groups by the intranasal combined intraperitoneal route were more severe than those by the single intranasal route.

The expression levels of the five cytokines all peaked at 14 dpi and decreased at 21 dpi. At 14 dpi, extremely significant differences in IL-1β (Fig. 1N) were observed between JS and J groups and between JS and 168 L groups. Compared with those induced by 168 L and PBS, JS induced a nearly 10-fold increase in IL-4 level (Fig. 1O). Compared with the PBS group, JS, J, and 168 L induced a 3.24-fold, 1.54-fold, and 1.37-fold increase in IL-6 levels, respectively (Fig. 1P). Compared with the PBS group, JS induced a 2.59-fold increase in TNF-α (Fig. 1O). JS induced the largest increase in IL-12 (Fig. 1Q) and had 22.07 times, 3.27 times, and 15.08 times than PBS, J, and 168 L, respectively; J induced an increase of 6.75 and 4.61 times than PBS and 168 L. The Th1/Th2 type of immune response induced mainly by Mhp also exhibits several inconsistencies.^3^ Proinflammatory cytokines have been measured in Mhp-infected pigs, and IL-12 was elevated.^5^ Here, IL-12 may be used as a criterion to distinguish between Mhp strains differing in virulence.

In summary, a BALB/c mouse infection model of respiratory mycoplasma was established, and the intranasal and intraperitoneal challenge route was used as the general method, which supports the evaluation of Mhp strain virulence and the elucidation of potential clinical diagnoses and pathogenic mechanisms of respiratory mycoplasmas not limited to Mhp.

## Ethics declarations

Animal experiments were approved by the Animal Experiment Ethics Committee (approval No: SYXK (Su) 2020–0024) of Jiangsu Academy of Agricultural Sciences according to guidelines of the Animal Management Regulations in Jiangsu Province (Government Order No. 45).

## Author contributions

X.X. conducted most of the experiments and wrote the manuscript; Y.G., M.D.P., L.Z., and F.H. helped with sample collection and gross lesion evaluation; Y.N.W. cultured mycoplasmas; Y.C., B.B.L., R.C., and Z.Z.Z. helped with flow cytometry analysis and real-time PCR; Q.Y. Xie helped with histopathological analysis; D.S. and G.Q.S. helped with the proinflammatory cytokine test; M.J.L., Q.Y. Xiong, and Z.X.F. conceived this study and contributed to the design and coordination. All authors agreed to and approved the final manuscript.

## Funding

This work was supported by the 10.13039/501100001809National Natural Science Foundation of China (No. 32273011, 32202812), 10.13039/100007540Jiangsu Agricultural Science and Technology Innovation Fund (No. CX(21)3124), Food Quality and Safety-State Key Laboratory Cultivation Base, Ministry of Science and Technology, China (No. 2022-SBGJZZ-6), and Jiangsu Association for Science and Technology Youth Science and Technology Talents Support Project (China) (No. JSTJ-2023-093).

## Data availability

Data and materials obtained and analyzed in this study are shown in this manuscript and supplementary file. Supplementary material related to this article can be found in the online version.

## Conflict of interests

The authors declared no conflict of interests.
