# Supplementary material for: Establishment of a mouse model of respiratory mycoplasma challenged intranasally combined intraperitoneally eliciting chronic inflammation and lung consolidation
Source: Genes Dis. 2024 Feb 9;12(1):101243. doi: 10.1016/j.gendis.2024.101243 (PMC12053559; doi:10.1016/j.gendis.2024.101243)
Supplement: Multimedia component 1 [file mmc1.docx]

**Supplementary methods/materials:**

This supplementary file shows the methods, tables and figures applied in supporting the results in the main text.

1. ***Experimental animals***

Ninety-two 5-week-old specific pathogen-free (SPF) female BALB/c mice (16–18 g) were obtained from the Animal Experiment Center of Yangzhou University. Antibody detection tests based on serologic testing demonstrated that the mice were free of the following pathogens: Mhp, Mp, Mo, *Mycoplasma hyorhinis*, influenza (H1, H3 subtype), porcine circovirus type 2, classic swine fever virus and other respiratory pathogens ^1,2,3^.

Germ-free mice, BALB/c mice and hamsters have been used to examine the role of cell-mediated immunity in *Mycoplasma pneumoniae* and *Mycoplasma pulmonis* infection ^4,5^. Female Swiss Webster mice ^6^ and BALB/c mice ^7^ have been used to provide preliminary evidence that *Mycoplasma genitalium* could induce genital tract infection. Few studies of Mhp and Mo have been performed in a murine model; for example, experiments by De Oliveira et al. ^8^ provided a chimeric protein evaluated in BALB/c mice that may serve as a candidate for the development of an Mhp vaccine. Another evaluation was performed by Bastola et al.^9^ in BALB/c mice for Mhp-specific antibody titres induced by inoculating adjuvants. Previous studies also investigated responses to inflammatory reactions and apoptosis in Mhp and Mo infection in murine alveolar macrophages^10,11^. Moreover, our recent studies demonstrated that BALB/c mice immunized with novel virulence factors of Mhp NFOR ^12^ and/or NOX ^13^, Th1 and Th17 mucosal immune responses were elicited by intranasal (IN) inoculation, especially intranasal and intramuscular (IN+IM) inoculation, in mice ^14^, indicating that multichannel injection can promote the improvement of immunogenicity. However, no murine respiratory model has been established for experimental infection by respiratory mycoplasmas of pig and goat species or for strain virulence assessment by Mhp.

Several studies have used mouse models infected with *Mycoplasma pneumoniae*, and germ-free mice and BALB/c mice have been the most commonly used to examine the role of cell-mediated immunity in *Mycoplasma pneumoniae*. To clarify the effects of respiratory Mp infection on airway fibrosis in asthma, Chu et al. ^15^ reported that double (2 wk apart) inoculations of Mp or saline (control) were given to BALB/c mice with or without previous allergen (ovalbumin) challenges. In addition, to find the pathological-radiological correlation of human *Mycoplasma pneumoniae* pneumonia, the literature regarding pathological and radiological studies of *Mycoplasma pneumoniae* and compared findings between open lung biopsy specimens and computed tomography (CT) were reviewed, and suggested that the pathological features of *M. pneumoniae* pneumonia may be altered by the level of host CMI, which highlights its advantages in radiological and pathological findings^16^.

Moreover, several studies have also used BALB/c mice to study inflammation induced by *M. pneumoniae* infection ^17-22^*.* These studies demonstrated that airway inflammation, bronchial hyperresponsiveness and pulmonary inflammation were induced by *Mycoplasma pneumoniae* infection in a murine model, and immunosuppression reduced lung injury caused by *Mycoplasma pneumoniae* infection. However, the disadvantages are that these studies cannot use a general inoculation method to induce lung consolidation by observing gross lung lesions after necropsy, which would simulate the lung lesions of *Mycoplasma pneumoniae* infection more intuitively and simply.

1. ***Respiratory Mycoplasma strains, growth conditions and antibodies***

The Mhp strains used here are JS (high-virulence strain), J (ATCC 25934), and 168 L (low-virulence strain), and the Mp and Mo virulent strains used are 129 (ATCC 29342) and IK3-3 (Biosample number of NCBI: SAMN29021347). Briefly, each Mhp strain was grown in modified Friis medium named KM2 cell-free medium containing 20% (v/v) pig serum that was sterilized by irradiation, and the bacteria were cultivated at 37 °C ^4^. Strain JS, a strain with high virulence, can lead to typical features of EP in conventional pigs, as we reported previously ^23^. Strain J (ATCC 25934) was passaged for three generations to obtain frozen stocks. Strain 168 L, a low-virulence strain, was attenuated by successive passages to the 352^nd^ generation from strain 168 with high virulence, which was isolated and cultured from a piglet with typical characteristics of mycoplasmal pneumonia in swine (MPS) from Gansu Province in Northwest China ^24^. The Mhp strain 168 L used herein was therefore at passage 355. For Mp strain 129 (ATCC 29342), which was reconstituted in SP4 broth and subcultured after 24 to 48 h in a flask containing 20 ml of SP4 medium at 37 °C until the broth turned an orange hue (approximately 72 h) and passaged for three passages before obtaining aliquots and stored at -70 °C^25^. Titres were tested and quantified with a 50% color change unit (CCU_50_) assay ^26^ that was modified from the CCU assay ^27^ with no contamination by other pathogens.

Goat anti-mouse IgG monoclonal antibody clone #RMG07 (Cat. No M04575-3) was purchased from Boster Bioengineering Co., Ltd. (Wuhan, China), and goat anti-mouse monoclonal antibody IgG1 (Cat. No A90–105AP) and goat anti-mouse monoclonal antibody IgG2a (Cat. No A90–107AP) were purchased from Bethyl Laboratories, Inc. (Montgomery, TX, USA). Mouse antibodies against CD3e-APC (Cat. No 17–0031–81), CD4-FITC (Cat. No 11–0041–81), and CD8a-PE (Cat. No 12–0081–81) were all purchased from EBioscience Inc. (Shanghai, China).

***Mouse infection, sample collection and experimental scheme***

A flow chart of mouse infection and sample collection scheme is shown in Figure 1. Every seventeen randomly selected mice inoculated with each Mhp strain JS, J, 168 L were treated as an independent experimental group, and mice treated with phosphate-buffered saline (PBS) were used as a negative control and were inoculated using the same methods as those used for mice in the Mhp-infected groups. At the same time, another twelve randomly selected mice were inoculated with Mp 129 and Mo IK3-3, which were treated as another two experimental groups. Each strain titre was set to 10^8^ CCU_50_/mL. Before inoculation, Zoletil (20 mg/kg, Virbac, Carros, France) was used to anaesthetize animals by intramuscular injection. Forty millilitres of each challenged strain was freshly cultured in KM2 medium (for Mhp and Mo strains) or SP4 medium (for Mp strain) at 0 and 2 days postinoculation (dpi). After centrifugation at 10 °C for 30 min at a speed of 10,000 rpm, each strain was concentrated to 4 mL in PBS, and 0.1 mL was used for CCU_50_ determination. For each Mhp-challenged group, at dpi 0 and 2, every 100 μL of the Mhp strain that was concentrated ten times was inoculated into seventeen anaesthetized mice in both nostrils (50 μL per nostril) intranasally (I.N.). Every mouse was then intraperitoneally (I.P.) injected with a total of 100 μL of the concentrated bacterial solution. For the Mp or Mo challenged group, 12 mice per strain were randomly divided into two parts and raised in separate cages. Half of the mice were I.N. inoculated with the abovementioned dose of both strains after anaesthetization, and the remaining mice were I.N.+I.P. injected with both strains as mentioned above.

For Mhp strains, five mice from each group were distinguished with ear tags. Mice were monitored for clinical symptoms, survival and body weight loss for three weeks. Although cough cannot be observed during the infection period, observation of the pleural respiratory rate through clinical symptoms can indirectly indicate oxygen uptake and lung function. Blood sample collection was performed at 7, 14 and 21 dpi, and necropsy was performed at 21 dpi. Two hundred microlitres of anticoagulant blood containing heparin sodium was collected from six mice per group for T lymphocyte subgroup detection, and 400 µL was collected for serum. Directly after necropsy, lung tissue from every mouse was first used to evaluate whether they demonstrated gross lesions, for example, whether consolidation appeared in the lung lobes. After pictures of lung tissues were taken immediately after necropsy, spleen and liver tissue were divided into two parts: half of the organ was weighed and stored for Mhp DNA copy real-time PCR testing, and the remaining part was used for histopathological examination. Lung tissue was divided into three parts taken from the gross lesions when they were present: the left lobes were weighed for real-time PCR, the right upper lobe was weighed for proinflammatory cytokine testing, and the right lower lobe was weighed for histopathological testing.

To see whether I.N.+I.P. The challenge route is a general route to other respiratory mycoplasmas. Mice inoculated with Mp 129 and Mo IK3-3 through the I.N. combined with or without the I.P. challenge route, lung gross lesions and pathological changes (lung, liver and spleen tissue) of mice infected with Mp and Mo strains were also verified. Many respiratory pathogens, including influenza virus and *Mycoplasma pneumoniae,* are pathogen infection models that can be better established by supplementation with the I.P. challenge route.

Previous studies have reported that combined challenge or immunization with different routes can promote the replication ability of various pathogens, such as bacteria, viruses or parasites, or promote the immune effect of corresponding vaccines. For example, the combined immunization (Flu/VV) with I.P.+I.N. route increased the number of gamma interferon-specific CD8 T cells by more than 60 times compared to that of the Flu/Flu scheme ^28^. I.P. inoculation with live influenza A virus confers protection against I.N. infections in mice and ferrets ^29^, and the I.P. & I.N. challenge route would be the most effective when live or ultraviolet-inactivated influenza viruses are introduced by different routes in a mouse model ^30^. Moreover, I.N., with and without sedation, and oral inoculation were compared with I.P. for establishing infection with a local isolate of Aleutian mink disease virus, and the I.P. & I.N. combined challenge method would be most ideal ^31^. Few studies on *Mycoplasma pneumoniae* infection in mice have demonstrated the distribution and reisolation of *M. pneumoniae* from various tissues of BALB/c mice that were I.P. preinoculated with Mp ^32^, and a recent study showed that I.P. inoculation was more efficient in disseminating infectious agents than I.N. and that the I.P. route would facilitate dissemination of infectious agents to the adventitia, which may be the main route for infectious agent entry into the circulation and many organs^33^. Moreover, human *Mycoplasma pneumoniae* (MP) pneumonia is characterized by alveolar infiltration with neutrophils and lymphocytes and lymphocyte/plasma cell infiltrates in the peribronchovascular area (PBVA). No mouse model has been able to mimic the pathological features seen in human MP pneumonia, such as plasma cell-rich lymphocytic infiltration in PBVA. To determine the mechanism for inflammation by MP infection, mice were preimmunized intraperitoneally with a Th2-stimulating adjuvant, alum, alone or MP extracts with an alum, followed by intratracheal challenge with MP extracts. These findings demonstrated that the acceleration of innate immunity by antecedent antigenic stimulation can be an important positive-feedback mechanism in lung inflammation during MP pneumonia ^34^. Although *Mycoplasma pneumoniae* pneumonia mainly occurs through upper respiratory tract infection, it is possible to at least partially, if not completely, simulate the invasion route, pathogenic range and severity of Mycoplasma by adding intraperitoneal injection.

Although oxygen uptake, pulmonary function, airway responsiveness, airway obstruction and other phenotypes were not detected in our present respiratory infection model, previous studies have demonstrated that airway inflammation, bronchial hyperresponsiveness and pulmonary inflammation were induced by *Mycoplasma pneumoniae* infection in a murine model, and immunosuppression reduces lung injury caused by *Mycoplasma pneumoniae* infection ^20-22,35-37^. Our mouse infection model is a chronic respiratory mycoplasma challenge model. Our main purpose is to establish a universal challenge method that can cause gross lung lesions to simulate infection in pigs and subsequently evaluate the effect of the corresponding mycoplasma vaccine. The mouse model will be supplemented with corresponding indicator measurements. Pictures of lung tissue from each mouse were taken, and gross lung lesions were carefully observed and evaluated. Bedding was changed every five days, and mice in each group were humanely killed at the indicated time points. Lung gross lesions were captured and observed after mice were infected with all respiratory Mycoplasma at 21 dpi. The histopathological pneumonia lesions from lung samples were evaluated following the criteria of lymphocyte infiltration and congestion or haemorrhage; the severity of the lesion was classified by scores in terms of 0 (normal), 1 (mild), 2 (moderate), and 3 (severe). Observers were unaware of the experimental groupings; moreover, they were qualified veterinary staff and could evaluate clinical symptoms ^3^.

Although in our present study, a mouse infection model was not involved in therapy with relevant anti-mycoplasma drugs, previous studies have been investigations on the *in vitro* and *in vivo* efficacy of ani-myboplasma drugs against Mycoplasma infection. For example, levonadifloxacin and nemonoxacin were confirmed to merit further study for treating infections caused by Mycoplasma infection^38,39^. For *in vivo* efficacy analysis, tigecycline treatment demonstrated a modest microbiologic effect, significantly improving lung histologic inflammation and reducing pulmonary cytokines and chemokines in a murine model of *M. pneumoniae* pneumonia^40^. In addition to tigecycline treatment, the immunomodulatory effect of clarithromycin in a murine model of lung inflammation induced by either live or UV-killed *Mycoplasma pneumoniae* was also evaluated ^35^. Moreover, resistance is inversely proportional to the presence and titre of *M. hominis*-specific serum antibodies. The possible role of cell-mediated immunity is discussed ^41^.

***Quantitative real-time PCR (qRT–PCR) of Mhp***

Mhp DNA was extracted from 0.1 g of lung, liver and spleen tissue homogenates in 1 mL sterilized PBS per mouse in a volume of 0.2 mL (Axygen, Cat. No: AP-MN-BF-VNA-250, NY, USA). qRT‒PCR assays were performed with primers and TaqMan-TAMRA probes designed based on the conserved sequence of the Mhp P97 gene ^23;24^. The samples of three tissues per group were analysed in six replicates.

***Anti-Mhp IgG antibody and IgG antibody subtype assessment***

Sera derived from every mouse at 14 and 21 dpi were tested for Mhp-specific antibodies (IgG, IgG1 and IgG2a) by indirect enzyme-linked immunosorbent assay (ELISA) ^1,42^. Briefly, whole-cell lysate proteins of strain 168 (GenBank Accession Number NC_017509.1, 4 μg protein/100 μL/well) were diluted and used to coat plates in carbonate ELISA coating buffer (0.05 mol/L, pH 9.6, including 2.93 g of sodium bicarbonate as well as 1.59 g of sodium carbonate dissolved in 1 L) at 4 °C overnight. Next, ELISA wash buffer (PBST, 0.05% Tween 20 dissolved in PBS) was used to wash the plate three times for 5 min per plate. Then, blocking buffer (PBS, pH 7.4, with 1% bovine serum albumin, 200 μL/well) was added to each well and incubated at 37 °C for 2 h. After three washes, 100 μL/well primary antibody (10 times dilution from each serum sample) was added before the plate was sealed and incubated at 37 °C for 1.5 h. After another triplicate washing, 100 μL/well diluted secondary antibody (goat anti-mouse HRP-conjugated IgG antibody with a dilution of 1:5000; goat anti-mouse alkaline phosphatase-conjugated IgG1 antibody or IgG2a antibody with a dilution of 1:1000) was added and incubated at 37 °C for another 50 min. After three washes, 100 μL/well TMB substrate solution (Cat. No P0209, Beyotime, Shanghai, China) or Alkaline Phosphatase Yellow Liquid Substrate System (Cat. No P7998, Sigma–Aldrich, Merck Life Science, Shanghai, China) was added, and the plate was placed in dark at room temperature (RT) for 15 min. Finally, 50 μL/well stop solution (0.5 M H_2_SO_4_ for IgG or 3 M NaOH for IgG1 or IgG2a) was added before measuring the optical density (OD) at 450 nm (IgG) or 405 nm (IgG1 or IgG2a) within 15 min, and the results are expressed as OD values. The cut-off was calculated as the mean OD value of mouse negative serum. The mouse serum samples collected from each group were analysed in six replicates.

***T lymphocyte subgroup detection of mouse anticoagulant peripheral blood***

The T lymphocyte subgroup detection method was performed as reported previously ^43^. Anticoagulated peripheral blood (100 μL) was collected in separate 1.5 mL EP tubes from each mouse in three Mhp-infected groups and one control group (six mice in each group) at 14 dpi and stained with the mouse monoclonal antibodies CD3-APC, CD4-FITC, and CD8-PE (0.25 μg/test, all from eBioscience) for 25 min at RT, and simultaneously, single-labelled groups with three antibodies individually labelled were also set up. Red blood cell lysis buffer (Cat No. QYR042, Fcmacs, Nanjing, China) was subsequently added to 2 μL (10 times dilution with PBS), and the cells were mixed gently by flicking the bottom of the tube, which was kept at RT in the dark for 15–30 min until the liquid became clear and transparent. The tubes were centrifuged at 4 °C for 5 min at 1000 rpm, the pellet was resuspended in 1 mL of PBS, and after two washes, the cells were resuspended in 200 μL of PBS. Then, 20 μL of 4% paraformaldehyde was added to the final suspension, and the tubes were placed at 4 °C overnight one day before use. A total of 2 × 10^4^ lymphocytes were analysed with a BD flow cytometer (BD FACSCalibur, NY, USA). The results are expressed as percentages of CD3^+^CD4^+^ T cells as well as CD3^+^CD8^+^ T cells in the total lymphocyte population in six replicates.

***Histopathological analysis***

After necropsy, lung, liver and spleen samples from each mouse in the Mhp or PBS groups (at 14 and 21 dpi) and in the Mp and Mo groups at 21 dpi were obtained, and pathological evaluation was performed as described previously ^3^.

***Determination of proinflammatory cytokine expression levels in lung homogenates***

Sections of the lungs (right upper lobes after weighing) were homogenized in 1 mL of PBS per gram of tissue. After centrifugation at 8,000 rpm for 10 min, the supernatants of homogenates were assayed for mouse tumour necrosis factor (TNF-α, Cat. No JYM0218Mo), interleukin 4 (IL-4, Cat. No JYM0011Mo), IL-6 (Cat. No JYM0012Mo), IL-12 (Cat. No JYM0550Mo), and IL-1β (Cat. No JYM0531Mo) using ELISA kits (Colourful Gene Biological Technology Co., Ltd., Wuhan, China).

***Statistical analysis***

All experiments were reproducible and carried out in triplicate, and all data are expressed as the mean±SD. Differences in serum antibody titres, body weight loss, lung cytokine levels, CD3^+^CD4^+^ T-cell lymphocyte positive percentages, variations in the number of DNA copies per gram of tissues between each Mhp group and control group, lung histopathological scores between mice in infected groups (JS, J, 168 L, 129 and IK3-3) and control group (PBS), and between two I.N. & I.P. and two I.N. methods were assessed through one-way analysis of variance (ANOVA) followed by Tukey’s multiple-comparison test for post hoc analysis using SPSS Statistics v20.0 and GraphPad Prism 8.3 software. *P* < 0.05 was considered a significant difference, and *P* < 0.01 was considered an extremely significant difference.

**References**

1. de Oliveira NR, Jorge S, Gomes CK, et al. A novel chimeric protein composed of recombinant *Mycoplasma hyopneumoniae* antigens as a vaccine candidate evaluated in mice. Vet Microbiol. 2017;201:146-153.
2. Kiupel M, Stevenson GW, Choi J, Latimer KS, Kanitz CL, Mittal SK. Viral replication and lesions in BALB/c mice experimentally inoculated with porcine circovirus isolated from a pig with postweaning multisystemic wasting disease. Vet Pathol. 2001;38(1):74-82.
3. Xie X, Na W, Kang A, et al. Comparison of the virulence of three H3N2 canine influenza virus isolates from Korea and China in mouse and Guinea pig models. BMC Vet Res. 2018;14(1):149.
4. Bowden JJ, Schoeb TR, Lindsey JR, McDonald DM. Dexamethasone and oxytetracycline reverse the potentiation of neurogenic inflammation in airways of rats with Mycoplasma pulmonis infection. Am J Respir Crit Care Med. 1994;150(5 Pt 1):1391-1401.
5. Hayakawa M, Taguchi H, Kamiya S, et al. Animal model of Mycoplasma pneumoniae infection using germfree mice. Clin Diagn Lab Immunol. 2002;9(3):669-676.
6. McGowin CL, Spagnuolo RA, Pyles RB. *Mycoplasma genitalium* rapidly disseminates to the upper reproductive tracts and knees of female mice following vaginal inoculation. Infect Immun. 2010;78(2):726-736.
7. Furr PM, Taylor-Robinson D. Factors influencing the ability of different mycoplasmas to colonize the genital tract of hormone-treated female mice. Int J Exp Pathol. 1993;74(1):97-101.
8. de Oliveira NR, Jorge S, Gomes CK, et al. A novel chimeric protein composed of recombinant *Mycoplasma hyopneumoniae* antigens as a vaccine candidate evaluated in mice. Vet Microbiol. 2017;201:146-153.
9. Bastola R, Seo JE, Noh G, et al. Determination of *Mycoplasma hyopneumoniae*-Specific IgG, IgG1, and IgG2a titers in BALB/c Mice induced by mineral oil-based oil-in-water emulsion adjuvants prepared using a self-emulsifying drug delivery system. AAPS PharmSciTech. 2019;20(1):31.
10. Damte D, Lee SJ, Birhanu BT, Suh JW, Park SC. Sonicated protein fractions of *Mycoplasma hyopneumoniae* induce inflammatory responses and differential gene expression in a murine alveolar macrophage cell line. J Microbiol Biotechnol. 2015;25(12):2153-2159.
11. Chen J, Zhou Y, Zhu E, et al. *Mycoplasma ovipneumoniae* induces caspase-8-dependent extrinsic apoptosis and p53- and ROS-dependent intrinsic apoptosis in murine alveolar macrophages. Virulence. 2021;12(1):2703-2720.
12. Xie X, Hao F, Chen R, et al. Nicotinamide adenine dinucleotide-dependent flavin oxidoreductase of *Mycoplasma hyopneumoniae* functions as a potential novel virulence factor and not only as a metabolic enzyme. Front Microbiol. 2021;12:747421.
13. Hao F, Xie X, Feng Z, et al. NADH oxidase of *Mycoplasma hyopneumoniae* functions as a potential mediator of virulence. BMC Vet Res. 2022;18(1):126.
14. Xu L, Hao F, Wang J, et al. Th1 and Th17 mucosal immune responses elicited by nasally inoculation in mice with virulence factors of *Mycoplasma hyopneumoniae*. Microb Pathog. 2022;172:105779.
15. Chu HW, Rino JG, Wexler RB, Campbell K, Harbeck RJ, Martin RJ. Mycoplasma pneumoniae infection increases airway collagen deposition in a murine model of allergic airway inflammation. American Journal of Physiology-Lung Cellular and Molecular Physiology. 2005;289(1):L125-L133.
16. Tanaka H. Correlation between radiological and pathological findings in patients with *Mycoplasma pneumoniae* pneumonia. Frontiers in Microbiology. 2016;7:8.
17. Saraya T, Nakata K, Nakagaki K, Kurai D, Takizawa H, Goto H. Identification of a mechanism for lung inflammation caused by *Mycoplasma pneumoniae* using a novel mouse model. Respirology. 2013;18:176-176.
18. Martin RJ, Chu HW, Honour JM, Harbeck RJ. Airway inflammation and bronchial hyperresponsiveness after *Mycoplasma pneumoniae* infection in a murine model. American Journal of Respiratory Cell and Molecular Biology. 2001;24(5):577-582.
19. Shi S, Zhang XQ, Zhou Y, Tang H, Zhao DY, Liu F. Immunosuppression reduces lung injury caused by *Mycoplasma pneumoniae* infection. Scientific Reports. 2019;9:8.
20. Hardy RD, Jafri HS, Olsen K, et al. *Mycoplasma pneumoniae* induces chronic respiratory infection, airway hyperreactivity, and pulmonary inflammation: a murine model of infection-associated chronic reactive airway disease. Infection and Immunity. 2002;70(2):649-654.
21. Chu HW, Honour JM, Rawlinson CA, Harbeck RJ, Martin RJ. Effects of respiratory *Mycoplasma pneumoniae* infection on allergen-induced bronchial hyperresponsiveness and lung inflammation in mice. Infect Immun. 2003;71(3):1520-1526.
22. Lin Y, Tan D, Kan QN, Xiao Z, Jiang ZY. The protective effect of naringenin on airway remodeling after *Mycoplasma pneumoniae* infection by inhibiting autophagy-mediated lung inflammation and fibrosis. Mediators of Inflammation. 2018;2018:10.
23. Gan Y, Xie, X., Zhang, L., Xiong, Q.Y., Shao, G.Q., Feng, Z.X. Establishment of a model of

*Mycoplasma hyopneumoniae* infection using Bama miniature pigs. Food Production, Processing and Nutrition. 2020;2(19):1-13.

1. Xie X, Hao F, Chen R, et al. Nicotinamide adenine dinucleotide-dependent flavin oxidoreductase of *Mycoplasma hyopneumoniae* functions as a potential novel virulence factor and not only as a metabolic enzyme. *Front Microbiol.* 2021;12:747421.
2. Hao Y, Kuang Z, Jing J, et al. *Mycoplasma pneumoniae* modulates STAT3-STAT6/EGFR-FOXA2 signaling to induce overexpression of airway mucins. *Infect Immun.* 2014;82(12):5246-5255.
3. Leigh SA, Evans JD, Branton SL, Collier SD. The effects of increasing sodium chloride concentration on *Mycoplasma gallisepticum* vaccine survival in solution. *Avian Dis.* 2008;52(1):136-138.
4. Furr PM, Taylor-Robinson D. Factors influencing the ability of different mycoplasmas to colonize the genital tract of hormone-treated female mice. *Int J Exp Pathol.* 1993;74(1):97-101.
5. Gherardi MM, Najera JL, Perez-Jimenez E, Guerra S, Garcia-Sastre A, Esteban M. Prime-boost immunization schedules based on influenza virus and vaccinia virus vectors potentiate cellular immune responses against human immunodeficiency virus Env protein systemically and in the genitorectal draining lymph nodes. J Virol. 2003;77(12):7048-7057.
6. Gautam A, Park BK, Kim TH, et al. Peritoneal Cells Mediate immune responses and cross-protection against influenza A Virus. Front Immunol. 2019;10:1160.
7. Baek K, Maharjan S, Akauliya M, et al. Comparison of vaccination efficacy using live or ultraviolet-inactivated influenza viruses introduced by different routes in a mouse model. PLoS One. 2022;17(10):e0275722.
8. Farid AH, Hussain I. A comparison between intraperitoneal injection and intranasal and oral inoculation of mink with Aleutian mink disease virus. Res Vet Sci. 2019;124:85-92.
9. Lloyd LC, Cottew GS, Anderson DA. Protection against enzootic pneumonia of pigs: intraperitoneal inoculation with live LKR strain of Mycoplasma hyopneumoniae. Aust Vet J. 1989;66(1):9-12.
10. Damte D, Lee SJ, Birhanu BT, Suh JW, Park SC. Sonicated protein fractions of Mycoplasma hyopneumoniae induce inflammatory responses and differential gene expression in a murine alveolar macrophage cell line. J Microbiol Biotechnol. 2015;25(12):2153-2159.
11. Saraya T, Nakata K, Nakagaki K, Kurai D, Takizawa H, Goto H. Identification of a mechanism for lung inflammation caused by Mycoplasma pneumoniae using a novel mouse model. Respirology. 2013;18:176-176.
12. Hardy RD, Rios AM, Chavez-Bueno S, et al. Antimicrobial and immunologic activities of

clarithromycin in a murine model of Mycoplasma pneumoniae-induced pneumonia. Antimicrobial Agents and Chemotherapy. 2003;47(5):1614-1620.

1. Martin RJ, Chu HW, Honour JM, Harbeck RJ. Airway inflammation and bronchial hyperresponsiveness after Mycoplasma pneumoniae infection in a murine model. American Journal of Respiratory Cell and Molecular Biology. 2001;24(5):577-582.
2. Shi S, Zhang XQ, Zhou Y, Tang H, Zhao DY, Liu F. Immunosuppression reduces lung injury caused by Mycoplasma pneumoniae infection. Scientific Reports. 2019;9:8.
3. Xue G, Crabb DM, Xiao L, Liu Y, Waites KB. In Vitro activities of the Benzoquinolizine Fluoroquinolone Levonadifloxacin (WCK 771) and other antimicrobial agents against Mycoplasmas and Ureaplasmas in humans, including isolates with defined resistance mechanisms. Antimicrobial Agents and Chemotherapy. 2018;62(11):5.
4. Wang N, Liu WC, Zhou YH, Liu Y. In vitro activities of Nemonoxacin and other antimicrobial

agents against human Mycoplasma and Ureaplasmas isolates and their defined resistance mechanisms. Frontiers in Microbiology. 2019;10:9.

1. Salvatore CM, Techasaensiri C, Tagliabue C, et al. Tigecycline therapy significantly reduces the

concentrations of inflammatory pulmonary cytokines and chemokines in a murine model of *Mycoplasma pneumoniae* Pneumonia. Antimicrobial Agents and Chemotherapy. 2009;53(4):1546-1551.

1. Taylor-Robinson D, Furr PM. Further observations on the murine model of *Mycoplasma hominis* infection. Journal of Medical Microbiology. 2010;59(8):970-975.
2. Bastola R, Seo JE, Noh G, et al. Determination of *Mycoplasma hyopneumoniae*-Specific IgG, IgG1, and IgG2a titers in BALB/c Mice induced by mineral oil-based oil-in-water emulsion adjuvants prepared using a self-emulsifying drug delivery system. AAPS PharmSciTech. 2019;20(1):31.
3. Chen Y, Li X, Tian L, et al. Dynamic behavior of lymphocyte subgroups correlates with clinical outcomes in human H7N9 infection. J Infect. 2014;69(4):358-365.

**Supplementary Tables**

**Table S1** Incidence and percentage of mouse lung gross consolidation lesions occurred after twice challenge with single or combined routes at dpi 21.

| Inoculation method | PBS | Mhp JS | Mhp J | Mhp 168L | Mp 129 | Mo IK3-3 |
| --- | --- | --- | --- | --- | --- | --- |
| I.N. | / | 1/6 | / | / | 1/6 | / |
| I.N. & I.P. | / | 6/6 | 2/6 | / | 6/6 | 4/6 |

**Table S2** Average DNA copies per gram of tissues and detection rate from BALB/c mice infected or mock-infected with various strains of Mhp at dpi 21*.*

| Group | DNA copies per gram of mouse lung tissues | DNA copies per gram of mouse liver tissues | DNA copies per gram of mouse spleen tissues |
| --- | --- | --- | --- |
| JS | 246694 ± 118099, 6/6 | 40183± 29642, 6/6 | 57107 ± 65705, 4/6 |
| J | 16502 ± 20696, 6/6 | 5881 ± 8464, 4/6 | 2115 ± 4855, 4/6 |
| 168L | 1096 ± 761, 5/6 | 24 ± 34, 3/6 | 0, / |
| PBS | 0, / | 0, / | 0, / |

**Supplementary Figures**

**Figure S1**

**
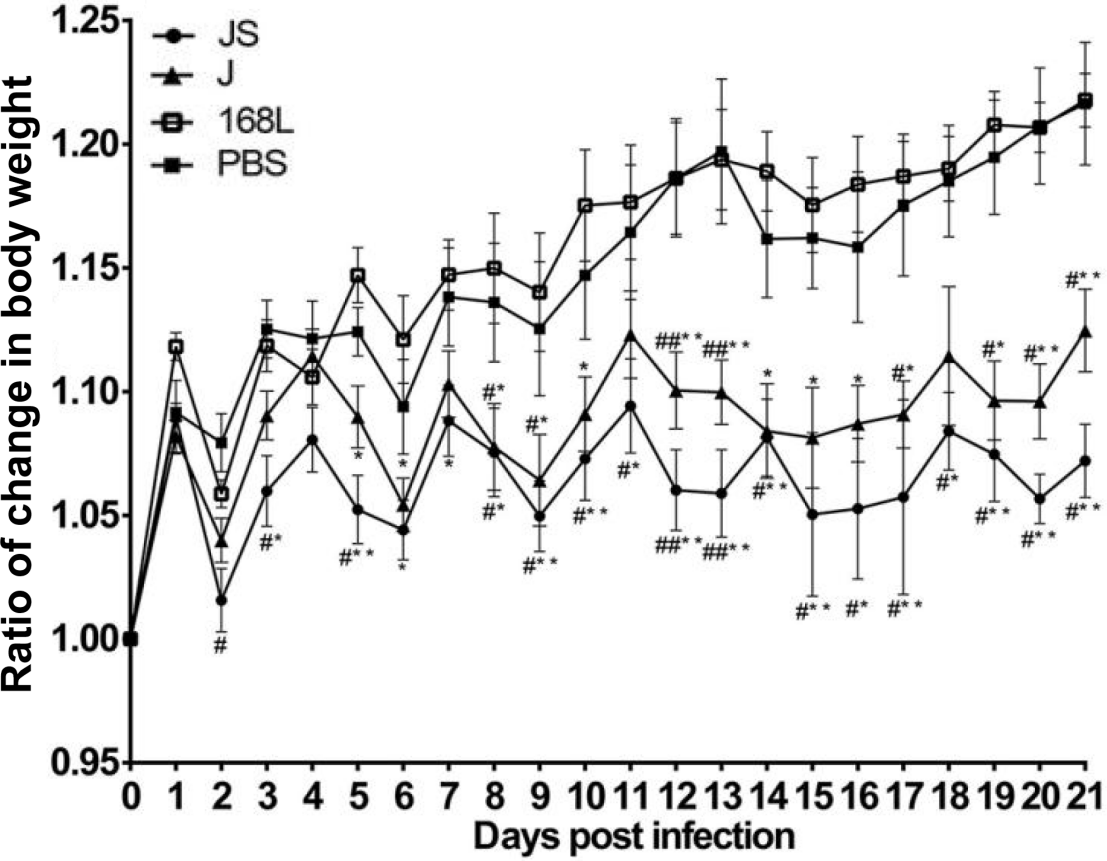
**

**Fig. S1 Body weight changes of mice infected with three Mhp strains.** Body weight loss was monitored and was expressed as ratio of change in body weight throughout 21 consecutive days. Error bars indicate the standard deviation (SD). *, *P* < 0.05 and **, *P* < 0.01 indicate significant mouse body weight changes between the JS and 168L groups or between the J and 168L groups. #, *P* < 0.05 and ##, *P* < 0.01 indicate significant differences in body weight changes between all infected groups and the PBS control group. Significant differences are depicted in the figure.


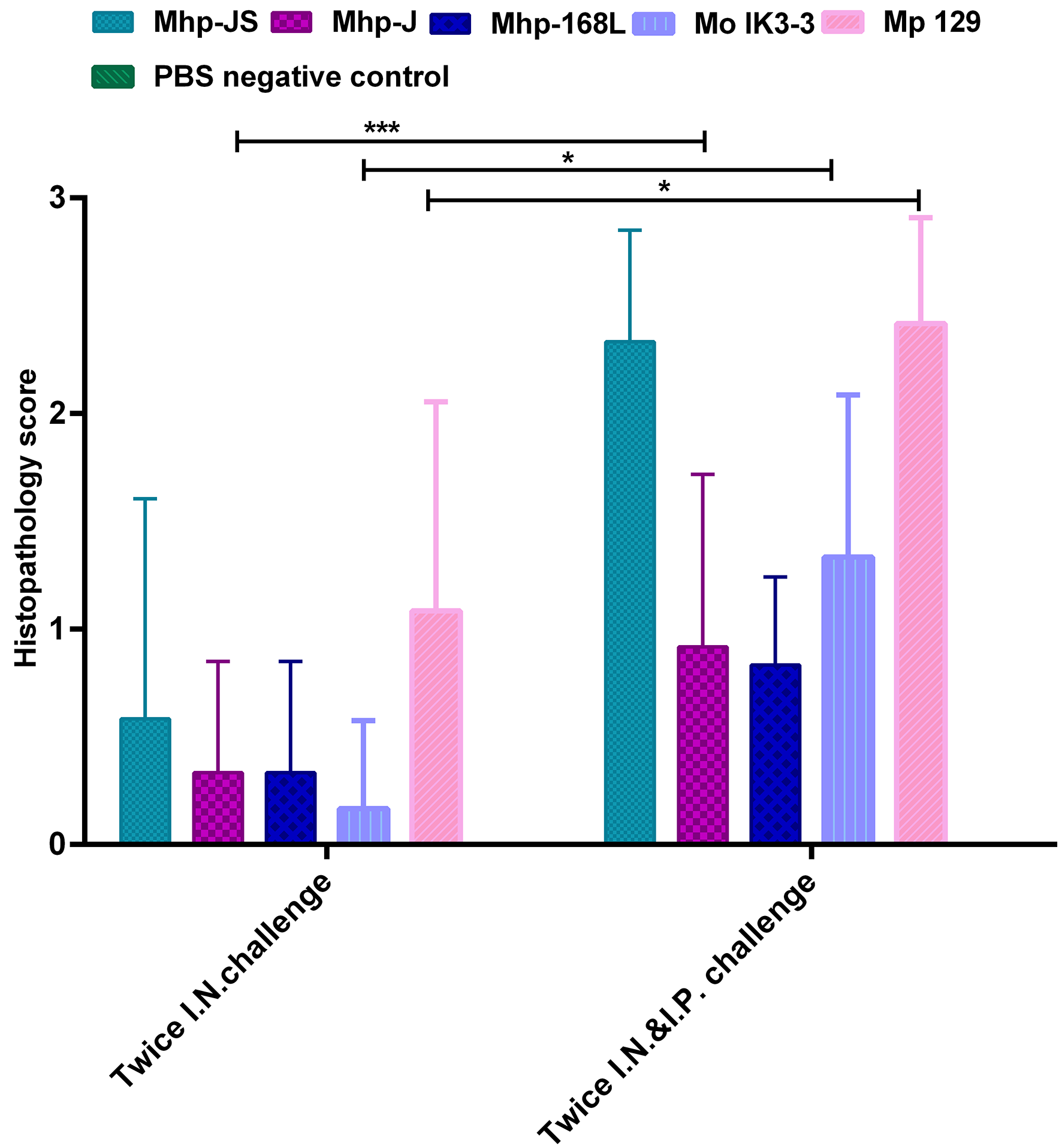


**Fig. S2 Histopathological assessment of lungs (B) from infected groups and PBS control group at 21 dpi through twice I.N. and twice I.N.+I.P. challenge mode.** *, *P* < 0.05, and ***, *P* < 0.01, indicates significant differences in the histopathological scores of lung tissues of mice in Mhp JS, Mo IK3-3 and Mp 129 groups through twice I.N. challenge and twice I.N. + I.P. challenge method, respectively. Data reflect the means ± SD of three independent experiments.
